# The Clinical Features and Emotional Stressors in Korean Patients with Tako-Tsubo Cardiomyopathy

**DOI:** 10.1155/2012/843876

**Published:** 2012-09-12

**Authors:** Bong Gun Song, Ju Hyeon Oh, Yong Hwan Park, Gu Hyun Kang, Woo Jung Chun

**Affiliations:** Division of Cardiology, Cardiac and Vascular Center, Department of Medicine, Samsung Changwon Hospital and Sungkyunkwan University School of Medicine, Changwon 630-522, Republic of Korea

## Abstract

*Background.* Tako-tsubo cardiomyopathy (TTC) is typically triggered by an acute emotional or physical stress events. Aim of this study was to investigate the impact of emotional stressors on clinical features, laboratory parameters, electrocardiographic and echocardiographic findings in patients with TTC. *Methods.* Of 103 patients enrolled from the TTC registry database, fifteen patients had emotional triggers (E group), and 88 patients had physical triggers or no triggers (other group). *Results.* Most clinical presentations and in-hospital courses were similar between the groups. However, E group had higher prevalence of chest pain (87 versus 42 %, *P* = 0.001), palpitation (27 versus 6%, *P* = 0.008), whereas other group had higher prevalence of cardiogenic shock (35 versus 7%, *P* = 0.027). E group had significantly higher corrected QT intervals (median, 477.5 versus 438 ms, *P* = 0.001), and left ventricular ejection fraction (LVEF) (mean, 45.7 versus 39.6%, *P* = 0.001), but lower hs-CRP (median, 0.1 versus 3.3 mg/L, *P* = 0.001), CK-MB (median, 5.5 versus 11.9 ng/mL, *P* = 0.047), troponin-I (median, 1.0 versus 3.2 ng/mL, *P* = 0.011), and NT-proBNP levels (median, 2145 versus 4939 pg/mL, *P* = 0.020). Other group required more frequent hemodynamic support and had significantly longer intensive care unit (median, 3 versus 1 days, *P* = 0.005) and in-hospital (median, 17 versus 3 days, *P* = 0.001) durations. *Conclusion.* The clinical features of TTC are different between groups with and without preceding emotional stressors. The TTC group with preceding emotional stressors was more likely to have preserved cardiovascular reserve and lesser likely to require hemodynamic support than other group although the entire prognosis of TTC is excellent regardless of triggering stressors.

## 1. Introduction

Tako-tsubo cardiomyopathy (TTC), also known as transient left ventricular (LV) ballooning syndrome, or stress-induced cardiomyopathy, is characterized by transient LV dysfunction in the absence of significant angiographic coronary stenoses, typically triggered by preceding emotional or physical stress [1–4]. It has been typically observed in postmenopausal females experiencing an emotionally stressful event inspiring to name this condition also broken heart syndrome and is increasingly being observed under diverse circumstances, including medical/surgical procedures or diagnostic test [5–7]. Differences in these clinical presentations have been observed, suggesting the possibility of diverse clinical phenotypes according to triggering stressors [6–8]. However, there have been few data to review and analyze the similarities and differences of clinical features between patients with TTC presenting with and those without preceding emotional stressors [9]. In this study, we investigated the impact of emotional stressors on clinical characteristics, laboratory parameters, electrocardiographic and echocardiographic findings in patients with TTC.

## 2. Materials and Methods

### 2.1. Study Subjects

We approached 103 consecutive patients enrolled from the TTC registry database at Samsung Changwon Hospital from January 2004 to December 2009. From 5078 consecutive patients with a diagnosis of an acute coronary syndrome, including ST- and non-ST-elevation myocardial infarction, who had an urgent coronary angiography (CAG), 103 (2%) patients were diagnosed with TTC. The enrolled 103 patients with TTC were divided into the following two subgroups according to the presence or absence of emotional stressors: fifteen (15%) patients had emotional triggers (E group), 88 patients had physical triggers or no triggers (other group). The criteria for inclusion were as follows: (1) transient akinesia/dyskinesia beyond a single major coronary artery vascular distribution, (2) absence of significant coronary artery disease on coronary angiograms (diameter stenosis < 50% by visual estimation) or angiographic evidence of acute plaque rupture, and (3) new electrocardiographic changes (ECG) (ST-segment changes, T-wave inversion, or Q-wave) [10]. Cardiogenic shock was defined as a systolic blood pressure < 90 mmHg for ≥30 minutes that was not responsive to fluid administration alone, accompanied by evidence of tissue hypoperfusion in the setting of clinically adequate or elevated LV filling pressures [11]. Pulmonary edema was defined as the presence of rales at pulmonary examination or a radiographic report of pulmonary alveolar/interstitial congestion at initial chest roentgenogram [11]. Hypertension was defined as repeated measurements of ≥140 mmHg SBP or ≥90 mmHg DBP, or previous antihypertensive drug treatment. Diabetes mellitus was defined as serum glucose level of 125 mg/dL or higher, a history of diabetes mellitus, or current use of anti-diabetic therapy. Current smoking was defined as having smoked cigarettes less than 1 year before patients presented with TTC. The protocol was approved by the Institutional Research Ethics Committee. The recommendations of the revised version of the Declaration of Helsinki were met.

### 2.2. Methods

The medical history and coronary risk factors were obtained from medical records combined with a patient questionnaire. Any physical or emotional stresses prior to the onset of this syndrome were specifically investigated. ECG and laboratory data including cardiac enzymes (creatine kinase [CK], creatine kinase MB fraction [CK-MB], and troponin-I) were recorded during the acute phase and were followed until the abnormalities disappeared. Followup data were collected by direct telephone interviews and a detailed review of all medical records. The cause and data of death were confirmed by information from the National Population Registry of the Korea National Statistical Office, together with a review of all available clinical records at the time of death.

### 2.3. Echocardiography

Transthoracic echocardiographic (TEE) examinations were performed in all patients with a 2.5 MHz transducer attached to a commercially available Doppler echocardiography machine, on the first hospital day or within 24 hours of CAG and followup. LV end diastolic (LVEDD) and end systolic diameters (LVESD), along with septal and posterior wall thickness at end diastole were measured in the parasternal long axis view, using 2D-guided M-mode echocardiography according to the recommendations of chamber quantification [12]. The LV ejection fraction (EF) was calculated by modified Simpson's method. The valvular regurgitation (VR) was assessed by color Doppler flow mapping of spatial distribution of the regurgitant jet in accordance with the ASE recommendation [13]. In our study, significant VR was defined as regurgitation of more than a mild degree. Reversible VR was defined as significant VR at initial echocardiogram that appeared at followup echocardiogram. Right ventricular systolic pressure (RVSP) was derived from tricuspid regurgitation gradient measured in accordance with the ASE recommendation [13]. In our study, we assessed the presence of systolic anterior motion (SAM) of the mitral valve using 2-dimensional imaging [14]. Left atrial (LA) volume was determined by the prolate ellipse method and indexed by body surface area (LAVI). 

### 2.4. N-Terminal Probrain Natriuretic Peptide (NT-proBNP) Assay

We took blood samples from the antecubital vein using lithium heparin, and the blood samples were then centrifuged. The blood samples were stored at −70°C until further analysis. Plasma NT-proBNP levels were measured using an Elecsys pro BNP reagent kit (Roche Diagnostics, USA) and an Elecsys 2010 (Roche Dignostics, USA). In all cases, the time interval between blood sampling for NT-proBNP and echocardiography was within 1 day.

### 2.5. Statistical Analysis

Statistical analyses were performed using SPSS statistical software (version 11.0, SPSS Corp, Chicago, IL, USA). Quantitative data are presented as mean ± standard deviation. Age, heart rate, corrected QT interval, hs-CRP levels, peak CK levels, peak CK-MB levels, peak troponin-I levels, left ventricular end diastolic pressure (LVEDP), duration of hospitalization, and duration of ICU stay are given in terms of the median and interquartile range (IQR). Qualitative data are presented as frequencies. The student's *t*-test or the Mann-Whitney test was used to compare the continuous variables and the chi-square test was used to compare the categorical variables. All *P* values are two tailed and differences were considered significant when the *P* value was less than 0.05.

## 3. Results

### 3.1. The Comparison of Clinical Characteristics between the Subgroups

**Figure 1:**
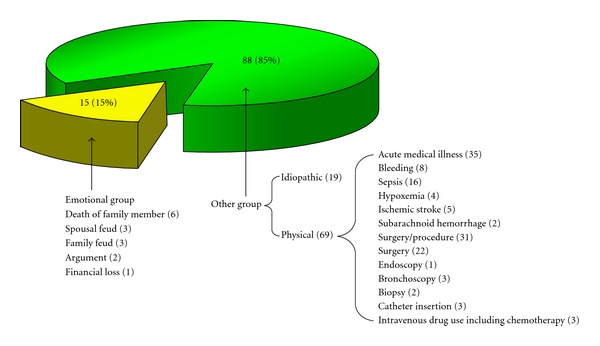
Preceding stressful events of 103 patients with TTC. Emotional stressors were documented in 15% of patients, whereas physical stressors were documented in 67% of patients and 18% of patients had no triggering stressor.

Emotional stressors were documented in 15% of patients (15/103), whereas physical stressors were documented in 67% of patients (69/103) and 18% of patients (19/103) had no triggering stressors (Figure 1). Among physical stressors, acute medical illness, surgery/procedure, and intravenous drug use were the precipitants in 34% of patients (35/103), 30% of patients (31/103), and 3% of patients (3/103), respectively (Figure 1). 

The clinical characteristics and initial presentations of the subgroups are compared in Table 1. The E group had significantly higher prevalence of chest pain (87 versus 42%, *P* = 0.001), and palpitation (27 versus 6%, *P* = 0.008), whereas other group had higher prevalence of cardiogenic shock (35 versus 7%, *P* = 0.027) than E group. The subgroups did not differ significantly in terms of age, male gender, body surface area, the prevalence of underlying diseases such as stroke, liver cirrhosis, hypertension, diabetes mellitus, and current smoking status. Also, there were no significant differences in clinical presentations such as dyspnea, loss of consciousness, nausea/vomiting, and pulmonary edema between two groups. 

### 3.2. The Comparison of ECG Changes, Laboratory, and Angiographic Findings between the Subgroups

 The ECG changes, laboratory, and angiographic findings of subgroups are compared in Table 2. The E group had significantly higher corrected QT interval (median, 477.5 versus 438.0 ms, *P* = 0.002), but lower heart rate (median, 84 versus 66 beats per minute, *P* = 0.001) than other group. The E group had significantly lower hs-CRP (median, 0.1 versus 3.3 mg/L, *P* = 0.001), peak CK-MB (median, 5.5 versus 11.9 ng/mL, *P* = 0.047), troponin-I (median, 1.0 versus 3.2 ng/mL, *P* = 0.011), NT-proBNP levels (median, 2145.0 versus 4939.0 pg/mL, *P* = 0.020), and LVEDP (median, 14 versus 19 mmHg, *P* = 0.039) than other group. However, there were no significant differences in ECG findings with regard to rhythm abnormalities such as atrial fibrillation, life-threatening arrhythmias, ST segment elevation, T-wave inversion, and Q-wave between two groups. 

### 3.3. The Comparison of Echocardiographic Findings between the Subgroups

The Echocardiographic findings of subgroups are compared in Table 3. The groups did not differ significantly in terms of ballooning patterns. The E group had significantly higher ejection fraction (mean, 45.7 versus 39.6%, *P* = 0.001), and lower LVEDD (mean, 49.5 versus 52.2 pg/mL, *P* = 0.009), and LVESD (mean, 37.9 versus 30.8%, *P* = 0.001) than other group. There were no significant differences in the LAVI, RVSP, and E/E′ on the initial and followup examinations between two groups. Also, there were no significant differences in SAM, significant mitral regurgitation (MR), aortic regurgitation (AR), and tricuspid regurgitation (TR) between two groups on the initial and followup examinations. All patients showed normalized regional wall motion in their followup echocardiogram. 

### 3.4. The Comparison of Management and Clinical Outcomes between the Subgroups

There were no significant differences in use of inotropics, use of intra-aortic balloon pump (IABP), use of angiotensin-converting enzyme inhibitor (ACEI) or angiotensin receptor blocker (ARB), and use of beta blocker during hospitalization between the two groups. The other group had significantly higher prevalence of diuretic use, and frequency of ICU stay and had significantly longer durations of in-hospitalization and ICU stay than the E group (Table 4).

During followup (median, 5.1 years, IQR, 4.0–6.1 years), 14 (14%) patients died; 8 patients died of malignancy, 2 of stroke, 2 of chronic renal failure with panperitonitis, 1 of liver cirrhosis with variceal bleeding, and 1 of pneumonia with empyema. However, cardiac deaths associated with LV ballooning syndrome itself were not noted in three groups. Also, recurrence of the LV ballooning syndrome was not noted in two groups. 

## 4. Discussion

TTC usually precipitated by an acute episode of emotional and/or physiological stress has been widely reported in the past [1–4]. Recently, diverse clinical features of TTC have been observed, suggesting the possibility of more than one clinical phenotypes according to triggering stressors [6–8]. However, a limited amount of literature currently exists on the similarities and differences of clinical features between patients with TTC presenting with and those without a preceding emotional stressors [9]. 

To the best of our knowledge, this is one of the largest studies investigating the similarities and differences of clinical features, laboratory parameters, electrocardiographic and echocardiographic findings between patients with TTC presenting with and those without emotional stressors. In the present study, E group had significantly higher prevalence of chest pain and palpitation, and higher corrected QT intervals and LVEF, whereas other group had significantly higher prevalence of cardiogenic shock and higher hs-CRP, CK-MB, troponin-I, and NT-proBNP levels. Other group required more frequent hemodynamic support and had significantly longer intensive care unit and in-hospital durations.

In contrast with the previously published data [5–8], our study showed that physical stress, rather than emotional stress, was the predominant triggering event for TTC. TTC cases associated with sepsis and respiratory failure without obstructive coronary artery disease were already reported [6–8], and several hypotheses have been proposed including catecholamine-mediated cardiotoxicity, spasm of the epicardial and/or microvascular coronary circulation, and endothelial cell dysfunction [15, 23, 24]. It was also surprising to know that TTC could occur during or after uneventful elective procedures or surgery performed under regional or general anesthesia. 

In the present study, most clinical features of E group were similar to those of other group. The overlapping clinical features in all these presentations may suggest that myocardial stunning resulting from emotional stress may share a common mechanism with those from physical stress, which has been described after subarachnoid hemorrhage and ischemic stroke which is believed to be mediated by catecholamine [15]. 

Our study showed that E group had significantly higher prevalence of chest pain and palpitation than other group. Some studies reported that catecholamine-mediated myocardial injury has been observed postmortem in people who died under terrifying circumstances such as violent assault, suggesting that catecholamine may be an important link between emotional stress and cardiac injury [15]. Therefore, we supposed that these subjective symptoms predominant in E group might reflect the emotional stressful event might trigger an excessive release of catecholamine, resulting in the presentations of these subjective symptoms and these patients appear to be more vulnerable to sympathetically mediated myocardial stunning or injury. 

Interestingly, in our study, other group had significantly higher prevalence of cardiogenic shock, but lower LVEF than E group. Also, other group had significantly higher hs-CRP, peak CK-MB, troponin-I, NT-proBNP, and LVEDP levels than E group. These observations may suggest that patients with idiopathic or physical stressors have more cardiovascular impairment and that perhaps other factors such as significant underlying comorbidities contribute to myocardial stunning or injury. The possible explanations may include myocardial stunning, inflammation, and microvascular spasm in idiopathic/physically stressed TTC patients [15, 16]. On the other hand, our patients with preceding emotional stressors had relatively preserved myocardial function with higher LVEF and lower LVEDP on initial presentation. These findings may suggest a transient catecholamine-induced myocardial stunning in this group. Therefore, we reasoned that patients with idiopathic or physical stressors had the greater extent of affected myocardium and cardiac markers such as CK-MB and troponin-I may reflect this extent of affected myocardium.

In contrast with recently published study showing that emotional stress group was younger than idiopathic/physical stress group [9], our data reported that the groups did not differ significantly in terms of age between two groups. The possible hypothesis for this discrepancy is that racial differences may affect emotional stressor and susceptibility of TTC with aging. Also, this discrepancy may be related to racial differences in activated sympathetic tones after a stressful event. 

A remarkable finding of our study was that sixty-seven (65%) of 103 patients had a prolonged corrected QT interval in the acute phases of TTC. Also, E group had significantly higher corrected QT interval, but lower heart rate than other group. These findings were similar to the findings of previous reports showing the associations between TTC and QT prolongation [17–20]. Seth et al. reported 12 cases of TTC with an average corrected QT interval of 478 ms [18]. Similarly, Abe et al. described 17 patients, most of whom had a prolonged corrected QT interval in the acute and subacute phases of the condition [19]. The QT interval normalized in all cases between 97 and 191 days from the onset of symptoms [19]. It is, therefore, reasonable to regard TTC as a transient acquired insult upon myocardial repolarization that may relate to an acute disturbance of cardiac autonomic function [17–21]. Based on these findings, we supposed that differences of these insults upon myocardial repolarization with triggering stressors may affect different QT intervals between the two groups. Further research is required to study our hypothesis about these differences. 

Notably, in our study, there were no differences in the use of inotropic agents, IABP, use of ACEI or ARB, and use of beta blocker between two groups. However, other group had significantly higher prevalence of diuretic use and frequency of ICU stay and had significantly longer durations of in-hospitalization and ICU stay than E group. Moreover, other group required more frequent hemodynamic support and had significantly higher prevalence of diuretic use, and significantly longer duration of hospitalization and ICU stay than E group. Based on findings of aforementioned studies [14, 22–24], in addition to the results of the present study, we reasoned that clinicians should monitor idiopathic/physically stressed TTC patients carefully for hemodynamic compromise. In our study, in-hospital and follow-up cardiac mortalities were 0%, respectively, in TTC patients with idiopathic/physical stressors. These findings may emphasize that the prognosis of TTC itself may be excellent if a meticulous therapeutic strategy under careful monitoring is performed in these patients, particularly in haemodynamic instability. 

In the present study, the overall mortality (16%) was relatively higher than in previous studies [1–4]. However, the overall mortality associated with TTC itself was 0%. It was comparable to results of published reports in other areas of the world [1–4]. During the follow-up period of 5.1 years, most patients (57%) died of malignancy in our study. According to previously published reports, malignancies may be associated with TTC, potentially as a result of paraneoplastic phenomena [25, 26]. These findings may indicate that patients with TTC have an excellent prognosis in the absence of significant underlying comorbidities such as malignancy or stroke. 

## 5. Study Limitations

There are some limitations that should be considered in our study. First, this was a retrospective analysis. Second, the results of our study may be limited by the relatively small number of patients. Third, we did not perform systemic investigations such as catecholamine measurements, magnetic resonance imaging, viral antibody titers, or pathology. Because TTC is a kind of exclusion diagnosis, some patients with other diseases, such as myocarditis, may be misdiagnosed in our study. However, diagnosis of TTC on the basis of clinical presentation and characteristic wall motion abnormality is realistic and often used in many studies. 

## 6. Conclusions

The clinical features of TTC are different between groups with and without preceding emotional stressors. The TTC group with preceding emotional stressors was more likely to have preserved cardiovascular reserve and lesser likely to require hemodynamic support than other group despite the entire prognosis of TTC is excellent regardless of triggering stressors. 

## Figures and Tables

**Table 1:** The comparison of clinical characteristics between the subgroups.

	Total (*n* = 103)	E group (*n* = 15)	Other group (*n* = 88)	*P*
Age (years)^†^	59.0 (52.0–72.0)	58.0 (54.0–78.0)	59.0 (52.0–72.0)	0.578
Male gender, *n* (%)	33 (32)	2 (13)	31 (35)	0.093
Body surface area (m^2^)	1.6 ± 0.2	1.5 ± 0.1	1.6 ± 0.2	0.093
Hypertension, *n* (%)	32 (31)	2 (13)	30 (34)	0.108
Diabetes mellitus, *n* (%)	22 (21)	2 (13)	20 (23)	0.412
Current smoker, *n* (%)	8 (8)	2 (13)	6 (7)	0.384
Underlying diseases				
Stroke/transient ischemic attack, *n* (%)	6 (6)	0 (0)	6 (7)	0.297
Liver cirrhosis, *n* (%)	2 (2)	0 (0)	2 (2)	0.555
Chronic renal failure, *n* (%)	7 (7)	0 (0)	7 (8)	0.258
Malignancy, *n* (%)	18 (17)	0 (0)	18 (20)	0.054
Clinical presentation				
Chest pain, *n* (%)	50 (49)	13 (87)	37 (42)	0.001*
Dyspnea, *n* (%)	54 (52)	9 (60)	45 (51)	0.525
Palpitation, *n* (%)	9 (9)	4 (27)	5 (6)	0.008*
Loss of consciousness, *n* (%)	1 (1)	0 (0)	1 (1)	0.678
Nausea/vomiting, *n* (%)	10 (10)	0 (0)	10 (11)	0.169
Cardiogenic shock, *n* (%)	32 (31)	1 (7)	31 (35)	0.027*
Pulmonary edema, *n* (%)	42 (41)	6 (40)	36 (41)	0.947

^†^Presented as median (interquartile range). *Significant finding.

**Table 2:** The comparison of electrocardiographic changes, laboratory, and angiographic findings between the subgroups.

	Total (*n* = 103)	E group (*n* = 15)	Other group (*n* = 88)	*P*
Electrocardiographic changes				
Life-threatening arrhythmia, *n* (%)	16 (16)	0 (0)	16 (18)	0.072
Heart rate (beats/min)^†^	80.0 (66.0–91.0)	66.0 (58.0–72.0)	84.0 (71.0–95.0)	0.001*
Atrial fibrillation, *n* (%)	6 (6)	0 (0)	6 (7)	0.297
Corrected QT interval (ms)^†^	438.0 (422.8–486.0)	477.5 (434.0–544.0)	438.0 (417.0–482.3)	0.002*
ST-segment elevation, *n* (%)	73 (71)	13 (87)	60 (68)	0.145
Q-wave, *n* (%)	13 (13)	0 (0)	13 (15)	0.111
T-wave inversion, *n* (%)	79 (77)	12 (80)	67 (76)	0.744
Laboratory findings				
hs-CRP (mg/L)^†^	2.3 (0.8–11.1)	0.1 (0.1–10.0)	3.3 (1.0–11.4)	0.001*
NT-proBNP (pg/mL)^†^	3210.0 (1102.0–30181.0)	2145.0 (613.3–5059.0)	4939.0 (1458.0–33017.0)	0.020*
Cardiac enzymes				
Peak CK (ng/mL)^†^	279.0 (93.0–632.0)	279.0 (519.0–619.5)	270.5 (90.0–607.0)	0.421
Peak CK-MB (ng/mL)^†^	11.9 (2.8–33.9)	5.5 (1.5–23.7)	11.9 (3.8–34.8)	0.047*
Peak troponin-I (ng/mL)^†^	2.0 (0.2–11.1)	1.0 (0.1–3.3)	3.2 (0.2–13.3)	0.011*
Angiographic findings				
LVEDP (mmHg)^†^	19.0 (13.0–25.0)	14.0 (9.0–20.0)	19.0 (13.0–25.0)	0.039*

Life-threatening arrhythmia: third-degree atrioventricular block, ventricular tachycardia, ventricular fibrillation, cardiac arrest; LVEDP: left ventricular end diastolic pressure.

^†^Presented as median (inter-quartile range). *Significant finding.

**Table 3:** The comparison of echocardiographic findings between the subgroups.

	Total (*n* = 103)	E group (*n* = 15)	Other group (*n* = 88)	*P*
*Initial TTE findings*				
Ballooning pattern				
Typical TTC pattern, *n* (%)	79 (77)	11 (73)	68 (77)	0.554
Inverted TTC pattern, *n* (%)	20 (19)	4 (27)	16 (18)	
Mid-ventricular pattern, *n* (%)	4 (4)	0 (0)	4 (5)	
Localized pattern, *n* (%)	0 (0)	0 (0)	0 (0)	
LVEF (%)	40.5 ± 8.0	45.7 ± 4.3	39.6 ± 8.2	0.001*
LVEDD (mm)	51.8 ± 6.1	49.5 ± 2.7	52.2 ± 6.9	0.009*
LVESD (mm)	36.9 ± 7.0	30.8 ± 4.8	37.9 ± 6.8	0.001*
LAVI (mL/m^2^)	28.2 ± 14.7	22.0 ± 3.8	29.3 ± 15.6	0.077
RVSP (mmHg)	33.7 ± 3.6	33.4 ± 3.2	33.8 ± 3.7	0.696
E/E′	10.8 ± 5.5	10.1 ± 2.9	11.0 ± 5.8	0.638
SAM	13 (13)	3 (20)	10 (11)	0.352
Significant MR, *n* (%)	25 (24)	5 (33)	20 (23)	0.376
Significant AR, *n* (%)	6 (6)	2 (13)	4 (5)	0.179
Significant TR, *n* (%)	15 (15)	4 (27)	11 (13)	0.151
*Follow-up TTE findings*				
LVEF (%)	62.9 ± 4.7	65.4 ± 5.1	62.5 ± 4.5	0.061
LVEDD (mm)	49.7 ± 5.3	50.4 ± 5.0	49.6 ± 5.4	0.664
LVESD (mm)	31.2 ± 4.2	29.6 ± 4.8	31.0 ± 4.1	0.187
LAVI (mL/m^2^)	27.6 ± 14.2	25.8 ± 3.4	27.7 ± 15.2	0.684
RVSP (mmHg)	31.8 ± 4.7	31.7 ± 4.7	32.0 ± 4.0	0.280
E/E′	10.5 ± 5.3	11.7 ± 3.1	9.5 ± 3.3	0.478
SAM	0 (0)	0 (0)	0 (0)	—
Significant MR, *n* (%)	9 (9)	2 (13)	7 (8)	0.495
Reversible MR, *n* (%)	16 (16)	3 (20)	13 (15)	0.605
Significant AR, *n* (%)	4 (4)	0 (0)	4 (5)	0.400
Significant TR, *n* (%)	9 (8)	1 (7)	8 (9)	0.224

TTE: transthoracic echocardiography; LVEF: left ventricular ejection fraction; LVEDD: left ventricular end-diastolic diameter; LVESD: left ventricular end-systolic diameter; LAVI: left atrial volume index; RVSP: right ventricular systolic pressure; E/E′: early diastolic mitral inflow velocity/early diastolic annular velocity; SAM: systolic anterior motion of anterior mitral leaflet; MR: mitral regurgitation; TRL: tricuspid regurgitation; AR: aortic regurgitation.

*Significant finding.

**Table 4:** The comparison of clinical courses and management between the subgroups.

	Total (*n* = 103)	E group (*n* = 15)	Other group (*n* = 88)	*P*
Use of inotropics, *n* (%)	28 (27)	2 (13)	26 (30)	0.192
Use of IABP, *n* (%)	12 (12)	0 (0)	12 (14)	0.128
Use of ACEI or ARB, *n* (%)	57 (55)	10 (67)	47 (53)	0.340
Use of beta blocker, *n* (%)	21 (20)	4 (27)	17 (19)	0.514
Use of diuretic, *n* (%)	40 (39)	2 (13)	38 (43)	0.028*
Temporal pacemaker, *n* (%)	10 (10)	0 (0)	10 (11)	0.169
Cardioversion, *n* (%)	6 (6)	0 (0)	6 (7)	0.297
ICU hospitalization, *n* (%)	63 (61)	5 (33)	58 (66)	0.017*
ICU hospitalization (days)^†^	2.0 (0–5.0)	1.0 (0–2.0)	3.0 (0–6.5)	0.005*
Hospitalization (days)^†^	14.0 (7.0–28.0)	3.0 (3.0–9.0)	17.0 (8.0–32.0)	0.001*
In-hospital cardiac mortality, *n* (%)	0 (0)	0 (0)	0 (0)	—
Cardiac mortality during followup, *n* (%)	0 (0)	0 (0)	0 (0)	—
Mortality during followup, *n* (%)	14 (14)	0 (0)	14 (16)	0.097
Recurrence, *n* (%)	0 (0)	0 (0)	0 (0)	—

IABP: intra-aortic balloon pump; ACEI: angiotensin converting enzyme inhibitor; ARB: angiotensin receptor blocker; ICU: intensive care unit.

^†^Presented as median (inter-quartile range).

*Significant finding.
